# Long Non-coding RNA Based Therapy for Cardiovascular Disease

**DOI:** 10.1007/s12265-025-10686-z

**Published:** 2025-09-03

**Authors:** Noelia Bellon Quinones, Ruggero Belluomo, Rio P. Juni, Reinier A. Boon

**Affiliations:** 1 Institute for Cardiovascular Regeneration, Centre for Molecular Medicine, Goethe University, Frankfurt Am Main, Germany; 2 German Centre for Cardiovascular Research, Partner Site Frankfurt Rhein/Main, Frankfurt, Germany; 3 Department of Physiology, Amsterdam University Medical Centers, Vrije Universiteit Amsterdam, Amsterdam, the Netherlands; 4 Amsterdam Cardiovascular Sciences, Microcirculation, Amsterdam, the Netherlands

**Keywords:** Long non-coding RNAs (lncRNAs), Cardiovascular diseases, Gene therapy, RNA therapeutics

## Abstract

Cardiovascular diseases (CVDs) remain a leading cause of morbidity and mortality worldwide, necessitating innovative therapeutic strategies. Long non-coding RNAs (lncRNAs) have emerged as regulators of gene expression, influencing various cellular processes involved in cardiovascular health and disease. This review explores the functional roles of lncRNAs in CVD pathogenesis, highlighting their involvement in processes such as hypertrophy, fibrosis, inflammation, and vascular remodeling. We discuss their potential as diagnostic biomarkers and therapeutic targets, alongside recent advancements in gene therapy approaches. While no lncRNA-based therapies have yet reached clinical trials, emerging RNA-targeting technologies, including antisense oligonucleotides, siRNAs, and CRISPR-based interventions, offer promising avenues for future therapeutic applications. Additionally, we examine the challenges associated with delivering lncRNA-based therapies, evaluating both viral and non-viral delivery methods, and their potential to revolutionize cardiovascular medicine. Ultimately, a deeper understanding of lncRNA biology and improvements in delivery strategies will be crucial in translating these findings into clinical treatments.

**Graphical Abstract:**

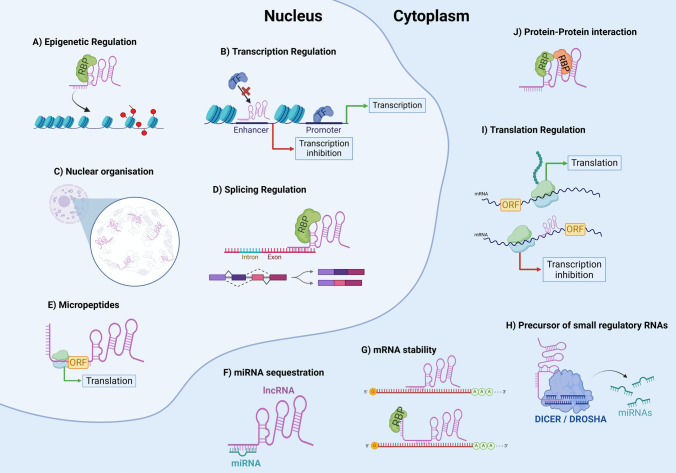

## Introduction of lncRNAs in CVD

Cardiovascular diseases (CVDs) encompass a wide range of disorders affecting the heart and blood vessels, including coronary artery disease, heart failure, arrhythmias, and hypertension. These conditions are leading causes of morbidity and mortality worldwide, with some offering limited treatment options, thus revealing the need for novel diagnostic and therapeutic strategies. Recent advances in molecular biology have highlighted the significant role of long non-coding RNAs (lncRNAs) in the pathogenesis and progression of CVDs, improving our understanding of the diseases and paving the way to novel therapeutic strategies targeting lncRNAs. However, despite the advancements, gaps remain in our understanding of lncRNAs in CVDs, which hinder their clinical application. In this review, we focus on how lncRNAs affect various cellular functions relevant for the progression and treatment of CVDs, and what challenges must be addressed to translate lncRNA-based therapies from bench to bedside, with a focus on delivery systems for the cardiovascular system.

### Overview on lncRNAs

Long non-coding RNAs (lncRNAs) are defined as transcripts longer than 200 nucleotides and that are not translated into proteins. Once considered transcriptional noise, lncRNAs are now recognized as critical regulators of various cellular processes. Furthermore, their discovery has transformed our understanding of gene regulation, challenging the traditional view that proteins are the sole regulators of genetic information [1]. Thousands of lncRNAs have already been identified in different species. In fact, human GENCODE suggests that more than 16,000 lncRNAs are present in humans alone, although others suggest that more than 100,000 lncRNAs may be present in humans [2, 3]. Most lncRNAs are transcribed by RNA Polymerase II and are processed similarly to mRNAs including capping, splicing, and polyadenylation.

One of the defining characteristics of lncRNAs is their remarkable diversity in genomic localization and regulatory mechanisms. Depending on their position relative to protein-coding genes, lncRNAs can be categorized into 5 classes: sense, anti-sense, intergenic, intronic, and bidirectional [1, 4, 5]. Sense lncRNAs are transcribed from the same DNA strand as adjacent protein-coding genes, often overlapping with an exon or spanning the entire sequence of a protein-coding gene. Anti-sense lncRNAs originate from the opposite strand of a protein-coding gene and can, for instance, interact with the corresponding mRNA by base-pairing and regulate its stability and translation. Intergenic lncRNAs are found between two protein coding genes and can function in *cis* or *trans* to regulate transcription, translation, and splicing. Intronic lncRNAs are usually found within introns of protein coding genes, they are generated by spliced introns, and they have been found to regulate the transcription of their host gene. Finally, bidirectional lncRNAs arise from the same promoter of protein-coding genes but in the opposite direction, and they can regulate chromatin accessibility and gene expression by interacting with the promoter region. lncRNAs localized between two protein-coding genes can be divided into two groups: promoter-associated and enhancer-associated lncRNAs (elncRNAs). Usually, elncRNAs regulate the expression of adjacent protein-coding genes, whilst promoter-associated lncRNAs regulate epigenetic inheritance and the state of the chromosome. Interestingly, despite their poor sequence conservation across species, many lncRNAs have conserved functions, suggesting that their secondary structure, rather than sequence, is crucial to their activity [6, 7].

**Fig. 1:**
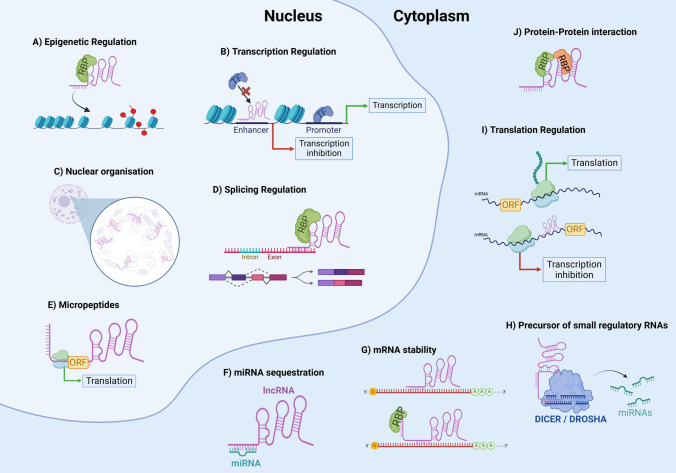
Long non-coding RNAs (lncRNAs) play diverse roles in gene regulation depending on their localization within the cell. The figure illustrates the multifaceted functions of lncRNAs, including (**A**) epigenetic regulation, where lncRNAs interact with chromatin-modifying complexes to influence DNA methylation and histone modifications; (**B**) transcription regulation, where lncRNAs modulate the activity of transcription factors to control gene expression; (**C**) nuclear organization, where lncRNAs contribute to the spatial arrangement of chromatin and the formation of nuclear substructures like nuclear speckles; (**D**) splicing regulation, where lncRNAs interact with splicing factors to influence alternative splicing events; (**E**) micropeptides, where some lncRNAs encode small functional peptides; (**F**) mRNA sequestration, where lncRNAs bind and sequester mRNAs to regulate their availability for translation; (**G**) mRNA stability, where lncRNAs interact with RNA-binding proteins to influence mRNA decay or stabilization; (**H**) precursor of small regulatory RNAs, where lncRNAs serve as sources for generating small RNAs that regulate gene expression; (**I**) translation regulation, where lncRNAs modulate the translation of target mRNAs; and (**J**) protein–protein interaction, where lncRNAs act as scaffolds or modulators to facilitate or inhibit interactions between proteins. These diverse roles highlight the critical involvement of lncRNAs in maintaining cellular homeostasis, directing development, and responding to environmental cues. These diverse roles highlight the growing importance of lncRNAs in cardiovascular disease research and their potential as therapeutic targets

In addition to their diverse genomic localization, lncRNAs also localize in different cell compartments, which contributes to their functional roles. As previously described, lncRNAs can affect transcription, mRNA translation and stability, and post-transcriptional processes. In particular, nuclear lncRNAs primarily regulate transcription and epigenetic changes, whereas cytoplasmic lncRNAs usually interact with mRNAs, miRNAs or proteins and regulate translation, stability, and localization (Fig. 1). Hence, the functions of lncRNAs can be broadly classified into three main categories: chromatin modification, transcriptional regulation, and post-transcriptional regulation.

Among the many function lncRNAs can elicit, epigenetic regulation is one of the most documented. In fact, the observations that lncRNAs are sequentially expressed in a spatio-temporal manner during different developmental stages led to the discovery that lncRNAs can modify chromatin accessibility by recruiting chromatin remodelling complexes to specific genomic regions [8]. For example, the well-known lncRNA HOTAIR was observed to negatively regulate the expression of the HOXD locus, which is part of the HOX gene cluster necessary for the development of tissues and organs during embryogenesis, by acting in trans to recruit the Polycomb chromatin remodelling complex PRC2 [8]. Furthermore, during X chromosome inactivation, the lncRNA Xist recruits PRC2 to inactivate the X chromosome, whereas the antisense lncRNA Tsix regulates PRC2 expression [9]. These studies highlight how lncRNAs can finely tune in gene expression in essential biological processes during developmental stages.

Besides regulating essential processes involved in developmental stages, lncRNAs can also regulate other cell processes such as cell death. In fact, lncRNAs regulate the expression of dihydrofolate reductase and guide heterologous nuclear ribonucleoprotein K to the promoter suppressed by p53, thus regulating cell response to cell death [10].

Given their multiple functions in essential cellular processes, it is not surprising that lncRNAs have been linked to numerous diseases. To date, a large number of lncRNAs has been linked to disease progression, including CVDs. In fact, more than 2000 lncRNAs are cardiac-specific, with 5 times higher expression in the heart than in other tissues. This highlights the importance of understanding lncRNA biology and their potential as therapeutic targets in diseases such as CVDs.

## LncRNA Targets in Cardiovascular Disease

- a) Key lncRNAs in Cardiovascular Health and Disease

In recent years, long non-coding RNAs (lncRNAs) have gained attention for their association with cardiovascular diseases (CVDs). Here, we summarize the roles of lncRNAs in CVDs (Table 1), including hypertension, atherosclerosis, aneurysms, atrial fibrillation (AF), myocardial infarction (MI), hypertrophic cardiomyopathy (HCM) and heart failure (HF). Further, we aim to highlight their potential in therapeutic applications. Although our understanding on their precise mechanisms of action remain incomplete, lncRNAs hold promise as novel diagnostic and treatment targets [11].

**Table 1:** lncRNAs involved in CVDs

	**Name**	**Category**	**Expression in disease**	**Target**	**Interaction**	**References**
**Hypertension**	AK098656	Intergenic	↑	α-SMA Fibronetin-1 MYH11	RNA–Protein interaction	[21]
	TUG1	Intergenic	↑	miR-145-5p	RNA-RNA interaction	[22]
	AK094457	Antisense	↑	PPARγ	RNA–Protein interaction	[23]
**Atherosclerosis**	TUG1	Intergenic	↑	miR-21	RNA-RNA interaction	[24]
	MeXis	Intergenic	↓	DDX17	Chromatin modification	[25]
	ANRIL	Antisense	Circular ANRIL↓ Linear ANRIL↑	CDKN2A/B	RNA–Protein interaction	[26, 27]
**Aneurysm**	HIF1A-AS1	Antisense	↑	Casp3/8, BRG1, BCL-2	RNA–Protein interaction	[28, 29]
	HOTAIR	Antisense	↓	Collagen types I/III	RNA–Protein	[30]
	H19	Intergenic	Discrepancies	miR-675, HIF1α, miR-148b, let-7a, MCP-1, Sp1, STAT3	RNA-RNA interaction, RNA–Protein interaction	[31–35]
**Atrial fibrillation**	TUG1	Intergenic	↑	miR-29b-3p	RNA-RNA interaction	[36, 37]
	MIAT	Intergenic	↑	miR-133a-3p	RNA-RNA interaction	[38]
	LIPCAR	Mitochondrial	↑	TGF-β/Smad	RNA–Protein interaction	[39, 40]
**Myocardial Infarction**	MIAT	Intergenic	↑	Furin/TGF-β/miR-24 miR-150	RNA–Protein interaction, RNA-RNA interaction	[41]
	Wisper	Intergenic	↑	TIA1-related protein Lysyl hydroxylase 2 (LH2)	RNA–Protein interaction	[42]
	Cfast	Possible intergenic* no data	↑	COTL1/TRAP1/TGF-ß	RNA–Protein interaction	[43]
	Sarrah	Antisense	↓	NRF2	RNA–Protein interaction	[44]
**Hypertrophic cardiomyopathy**	CHAIR	Intergenic	↓	DNMT3A	RNA–Protein interaction	[45]
	CHAST	Antisense	↑	PLEKHM1	RNA–Protein interaction	[46]
	Meg3	Intergenic	↑	P53 Mmp-2 promoter	RNA–Protein interaction	[47]
**Heart failure**	LncKCND1	Intronic	↓	YBX1	RNA–Protein interaction	[15]
	GAS5	Intergenic	↑	miR-21/PTEN	RNA-RNA interaction	[17]
	ZNF593-AS	Antisense	↓	HNRNPC	RNA–Protein interaction	[48]

One of the most relevant and widely studied lncRNAs in CVDs is MIAT, an intergenic lncRNA located on chromosome 22. It has been reported that this lncRNA plays a role in the development or progression of several CVDs, such as MI [12], atherosclerosis [13], and AF [14]. When MIAT is upregulated after a MI, it promotes fibrosis and, when silenced, it lowers fibrosis and partially recovers cardiac function. As a result, MIAT plays a critical role in cardiac remodeling in the context of MI. The molecular mechanisms by which MIAT regulates the fibrotic response following MI involve its binding to miR-24, which regulates TGF-β1 and Furin, two crucial fibrosis-related proteins [12]. Other lncRNAs are yet to be characterized and studied to improve the understanding of the initiation and progression of CVDs.

One example of a key lncRNA in CVDs is lncKCND1. This lncRNA is located in the X chromosome near the KCND1 gene, which is involved in voltage-gated K + channels, and it overlaps with the protein-coding gene GRIP1, an interacting protein of the glutamate receptors. lncKCND1 seems to be downregulated in hypertrophic mouse hearts (TAC model) and Angiotensin II (Ang II)-treated neonatal cardiomyocytes [15]. Lower expression of lncKCND1 is correlated with mitochondrial dysfunction in cardiomyocytes and consequently, an aggravation of the cardiac remodeling. The molecular mechanism underlying this process is through RNA–protein interaction. There are binding sites in the lncKCND1 to the YBX1 protein, leading to YBX1 upregulation. LncKCND1 overexpression inhibits hypertrophy, improves mitochondrial function, and protects the heart function in hypertrophic models, while its knockdown aggravates the pathological remodeling by lowering YBX1 expression. Similarly, silencing YBX1 also induces pathological remodeling, highlighting the functional interplay between lncKCND1 and YBX1 [16]. However, the majority of the evidence currently available comes from preclinical models, and additional research in human tissues is required to confirm these results and determine their translational significance.

While lncKCND1 appears to play a protective role in cardiac hypertrophy, other lncRNAs have been linked to distinct cardiovascular conditions. One such example is GAS5, a recently identified lncRNA involved in atrial fibrillation (AF). GAS5 expression is significantly downregulated in AF patients, both in circulating blood and atrial tissue, and this reduction occurs even before structural changes such as left atrial enlargement are evident. Functionally, GAS5 appears to suppress cardiomyocyte proliferation by targeting ALK5, suggesting a role in limiting fibrotic remodeling. Moreover, lower GAS5 levels have been associated with increased risk of AF recurrence after radiofrequency catheter ablation, highlighting its potential as both a mechanistic regulator and a prognostic biomarker for AF progression and therapeutic outcomes [17, 18]. Clinical data support its biomarker potential, but the mechanistic insights are still limited and call for more thorough functional studies in vivo.

Beyond arrhythmogenic processes, lncRNAs also play a central role in vascular aging, as shown by the recently characterized lncRNA MIRIAL [19] (10). Aging compromises the vascular homeostasis and enhance endothelial dysfunction and MIRIAL have recently been identified as an important regulator of it. MIRIAL, which was discovered by RNA-sequencing of aged cardiac endothelial cells, increases the expression of FOXO1 through an unusual mechanism for lncRNAs: the formation of a triplex structure at its promoter via an Alu element. Its knockdown in HUVECs impairs proliferation, migration, and basal angiogenic sprouting, while increasing mitochondrial activity and VEGFA stimulated responses. An animal model of MIRIAL deficiency reinforces its protective function in vivo by impairing cardiac function after myocardial infarction. MIRIAL is a promising therapeutic and diagnostic target for vascular aging and cardiovascular disease because of its involvement in a number of age-related conditions, such as aneurysms and ischemic heart disease [19]. Despite these innovative mechanistic findings, MIRIAL's function has not yet been confirmed in human clinical trials, and more investigation is required to elucidate its wider applicability across various types of CVD.

Similarly, the recently discovered lncRNA SMANTIS also utilizes an Alu-dependent mechanism to carry out its regulatory function. SMANTIS is a regulator of monocyte adhesion to endothelial cells, a key step in vascular inflammation and atherogenesis. SMANTIS is highly expressed in monocytes but downregulated during their differentiation into macrophages, and it interacts with the transcription factor RUNX1 via an Alu element. Loss of SMANTIS reduces RUNX1 genomic binding and alters its interaction with EP300 and CBFB, leading to impaired regulation of adhesion-related genes. These findings suggest that SMANTIS may control monocyte recruitment to the vascular wall, positioning it as a potential modulator of cardiovascular diseases such as atherosclerosis [20]. However, the evidence is still preliminary, and further studies are needed to validate its in vivo function and relevance in human pathology.

- b) Therapeutic Potential

Studies on lncRNAs, performed both in vitro and in vivo, reveal significant differences between cellular and whole-genome investigations, as well as variations across different cell types, highlighting the complexity of this research. Moreover, the mechanisms of action of lncRNAs are not yet fully understood. Due to their highly cell-specific expression, predominant nuclear localization, and diverse secondary structures, the mechanisms underlying lncRNA functions remain poorly understood [49].

When it comes to therapeutic applications, several challenges remain, including limited tissue-specific delivery, inefficient endosomal escape, and the additional barrier of tissue fibrosis (particularly in cardiovascular diseases) [50]. The delivery of lncRNAs to non-hepatic tissues, for example the heart, remains a major priority in lncRNA therapeutics. Recent research has shown that it is possible to increase the activity of oligonucleotides in extrahepatic tissues by employing small molecules that interfere with endolysosomal trafficking. This offers a promising approach to overcome one of the main delivery bottlenecks [51].

Nevertheless, a variety of delivery platforms are currently being explored to address the diverse requirements across different disease contexts: lipid nanoparticles (LNPs), exosome-based carriers and viral vectors among others [50], which will be discussed further in this review. Choosing between viral vectors (e.g., adenovirus, lentivirus) and non-viral systems is especially relevant when targeting cardiomyocytes or when sustained expression is required.

As delivery technologies improve, understanding how lncRNAs regulate gene expression epigenetically in cardiovascular pathology is crucial for developing more accurate, efficient RNA-based therapies that minimize off-target effects [52]. Even with these limitations, the research on lncRNA shows great promise due to their cell-type specificity and the potential to target specific cardiovascular phenotypes by regulating their expression. Modulating lncRNA expression can be achieved using tools such as adenoviruses, lentiviruses, or RNA-based methods like siRNA, aptamers, and GapmeRs [49].

Currently, RNA-based therapies are being tested in humans through clinical trials. Among these, targeting hyperlipidemia as a preventive strategy has gained significance for its implications in the onset of CVDs like atherosclerosis [53]. One example is Inclisiran, an RNA interference (RNAi) based-drug (siRNA), which has already been approved by FDA and EMA. Inclisiran helps the liver to remove LDL cholesterol from the bloodstream more efficiently by lowering the proprotein convertase subtilisin–kexin type 9 (PCSK9), which stops the liver cells'LDL receptors from being destroyed. A subcutaneous injection significantly reduces PCSK9 and LDL levels, maintaining these effects for over 6 months [54]. Similar to Inclisiran, Zodasiran is another siRNA based-drug targeting hyperlipidemia. This drug, currently in phase II clinical trials, targets angiopoietin-like 3 (ANGPTL3), leading to loss of its function. Consequently, lipoprotein lipase (LPL) and endothelial lipase become more available in the organisms, breaking down triglycerides and other lipids in the bloodstream, which improves hyperlipidemia condition [55]. Following this strategy of targeting ANGPTL3, Vupanorsen, an antisense oligonucleotide (ASO), has been developed to also target ANGPTL3 mRNA in the liver, leading to similar effects as Zodasiran, and has passed phase II of clinical trials [56]. However, despite meeting its primary objectives, drug development was discontinued in January 2022 due to the insufficient efficacy and liver toxicity.

Despite the challenges in fully understanding lncRNA processes, their promise in cardiovascular research continues to grow, especially in the development of targeted therapies. Although no lncRNA-based therapeutics are currently undergoing clinical trials, insights gained from successful RNA-based therapies could pave the groundwork for future lncRNA-targeting treatments. As clinical trials advances, these approaches may lead to more accurate and efficient therapies, which eventually improve patient outcomes. To take full advantage of the therapeutic potential of lncRNAs, future research should focus on deepening our understanding of their interactions and enhancing delivery strategies. Additionally, improving delivery strategies will be key to translating this knowledge into clinical applications.

## Gene Therapy Delivery Methods

Currently, 2 types of delivery methods for gene therapies are available, namely viral and non-viral vectors. Viral vectors are composed of non-pathogenic viral envelopes with packaging machinery and capsids, while non-viral delivery systems such as lipid nanoparticles (LNPs) generally consist of ionizable lipids, amphipathic phospholipids, and cholesterol, which encapsulate nucleic acids and facilitate their delivery into the cells. While several gene therapies, including miRNA mimics and antimiRs, are currently undergoing clinical trials or already in the market (Table 2), no therapeutics based on lncRNA have yet reached this phase. Nevertheless, it is plausible that the same delivery methods can be utilized for lncRNA-based therapies in the future.

**Table 2:** Gene Therapies in Clinical Trials or Approved by FDA/EMA for CVDs

Clinical trial ID	Disease	Target gene	Approach	Therapy	Delivery method	Clinical trial phase
Genetic cardiomyopathy						
NCT06109181	**Arrhythmogenic cardiomyopathy**	**PKP2**	**Gene replacement**	**LX2020**	**AAV**	**2**
NCT05836259	**Hypertrophic cardiomyopathy**	**MYBPC3**	**Gene replacement**	**TN-201**	**AAV**	**1b**
Heart failure						
NCT01966887	**Non-ischemic cardiomyopathy**	**SERCA2a**	**Gene replacement**	**MYDICAR**	**AAV**	**2 (terminated)**
NCT05598333	**Ischemic cardiomyopathy**	**PP1**	**Protein inhibition**	**AB-1002**	**AAV**	**2**
NCT03409627	**Outpatient LVAD**		**Gene delivery**	**INXN-4001**	**Plasmid**	**1**
Hyperlipidemia						
NCT00891306	**Familial hypercholesterolemia**	**LDLR**	**Gene replacement**	**LPLS447X**	**AAV**	**3**
NCT06112327	**Familial hypercholesterolemia**	**PCSK9**	**Gene editing**	**VERVE-101**	**LNP**	**1**
NCT05860569	**Hypertriglyceridemia**	**LPL**	**Gene replacement**	**GC304**	**AAV**	**1**
Inclisiran	**Primary hypercholesterolaemia**	**PCSK9**	**RNA interference**	**Leqvio**	**siRNA**	**FDA-EMA approved**
NCT04832971	**Mixed dyslipidemia**	**ANGPTL3**	**RNA interference**	**ARO-ANG3 (Zodasiran)**	**siRNA**	**2b**
NCT03070782	**Hyperlipoproteinemia(a)**	**LPA**	**RNA interference**	**APO(a)-LRX**	**ASO**	**2a**
NCT04270760	**Hyperlipoproteinemia**	**LPA**	**RNA interference**	**Olpasiran**	**siRNA**	**2**
Muscular dystrophy						
NCT05689164	**Duchenne muscular dystrophy**	**DMD**	**Gene replacement**	**AAV9-minidystrophin**	**AAV**	**3**
NCT02354781	**Duchenne muscular dystrophy**	**DMD**	**Myostatin inhibitor**	**AAV-follistatin**	**AAV**	**2**
NCT06392724	**Duchenne muscular dystrophy**	**DMD**	**Base editing/exon skipping**	**GEN6050X**	**AAV**	**1**
Golodirsen	**Duchenne muscular dystrophy**	**DMD**	**Exon skipping**	**Vyondys**	**ASO**	**FDA-EMA approved**
Genetic syndrome						
NCT04601051	**Transthyretin amyloidosis**	**TTR**	**Gene editing**	**NTLA-2001**	**LNP**	**1**
NCT05445323	**Friedrich ataxia**	**FXN**	**Gene replacement**	**LX2006**	**AAV**	**2**
NCT03533673	**Pompe disease**	**GAA**	**Gene replacement**	**ACTUS-101**	**AAV**	**2**
NCT03454893	**Fabry disease**	**GLA**	**Gene replacement**	**AVR-RD-01**	**Lentivirus**	**2 (terminated)**
NCT04046224	**Fabry disease**	**GLA**	**Gene replacement**	**ST-920**	**AAV**	**2**
NCT06092034	**Danon disease**	**LAMP2**	**Gene replacement**	**RP-A501**	**AAV**	**2**
NCT00454818	**Dilated cardiomyopathy**	**SERCA2a**	**Gene replacement**	**Cupid**	**AAV**	**2**
NCT01064440	**Critical limb ischemia**	**HGF**	**Gene delivery**	**VM202**	**Plasmids**	**2**
NCT02544204	**Peripheral Artery Disease**	**SDF1**	**Gene delivery**	**JVS-100**	**Plasmids**	**2**

### Viral Vectors

#### Adenoviruses

Adenoviruses (AVs) are a widely used platform for gene therapy, due to their ability to transduce both dividing and non-dividing cells. These non-enveloped, double-stranded DNA viruses possess a large cargo capacity of up to ~ 8 kb, making them suitable to deliver large transgenes which may include their regulatory elements such as promoters and enhancers [57]. Recombinant AVs are engineered to remove pathogenic viral genes, such as E1 and E3, to prevent replication and promote safety. They also enable the use of tissue-specific promoters and capsid modifications to improve targeting of specific tissues or cell types [57]. A key advantage of AVs is their episomal nature, where their genetic material does not or minimally integrate into the host genome, which minimizes risks of insertional mutagenesis and ensures transient expression [58, 59]. Coupled with their broad tropism and rapid onset of expression, AVs are ideal for applications that require short-term therapeutic effects [60].

Besides their advantages, AVs also present notable challenges. Their strong immunogenicity triggers robust innate and adaptive immune responses, limiting their suitability for dose repetition and increasing the risk of inflammation and toxicity at high doses [61]. Additionally, the transient nature of AV-mediated expression may limit their utility for chronic conditions that require sustained gene activity. Pre-existing immunity to certain AV serotypes further complicates their therapeutic use, as neutralizing antibodies can reduce efficacy. The strategies to overcome these limitations include the development of "gutless" or high-capacity AVs, which delete all viral coding sequences to reduce immunogenicity and the use of synthetic capsids to evade pre-existing immunity and improve tissue specificity [62].

#### Adeno-associated Viruses

Adeno-associated viruses (AAVs) are commonly used vectors for gene therapy due to their safety profile, long-term gene expression capabilities, and tissue-specificity. AAVs are small, non-enveloped, single-stranded DNA viruses, which belong to the parvovirus family, that do not cause human disease and minimally integrate into the host genome, reducing the risk of insertional mutagenesis [63].

AAVs are inherently non-pathogenic and elicit only mild immune responses, making them ideal for in vivo applications [64]. Unlike AVs, AAVs rarely provoke severe inflammatory responses, even at high doses [65]. AAVs achieve long-term expression of the transgenes due to their ability to persist in an episomal form and to integrate into the host genome at specific sites in host cells. This integration, albeit in very low levels, can contribute to more stable gene expression as compared to Avs [66]. This feature is particularly useful in cardiac applications, where sustained gene expression is needed to address chronic conditions such as heart failure. Further, AAVs exhibit diverse serotypes that allow for tissue-specific targeting. AAV9 is known for its high tropism to cardiac cells, particularly, cardiomyocytes, making it a preferred serotype for cardiac therapies [67]. Modification of the capsid may lead to higher specificity towards other cardiac cell types, such as endothelial cells [68].

One of the most prominent applications of AAVs in the cardiovascular field was the Calcium Upregulation by Percutaneous Administration of Gene Therapy in Cardiac Disease (CUPID) clinical trials. In these studies, AAV1 was used to introduce sarcoplasmic reticulum Ca^2^⁺ATPase (SERCA2a), into cardiomyocytes, aiming to restore calcium handling and improve cardiac function in patients with heart failure with reduced ejection fraction. Although the first trial showed promising results, the CUPID II trial showed insufficient therapeutic efficacy and adverse immune responses, highlighting the complexities of implementing gene therapy in clinical settings [69]. This trial did not meet its primary endpoint (time to recurrent events) or its secondary endpoint (time to first terminal event). A possible explanation for this outcome is the inadequate delivery of the vector genome to cardiomyocytes. Although patients enrolled in the trial did not show detectable levels of neutralizing antibodies against AAV1, even the sample with the highest AAV genome copy number revealed that less than 2% of cardiomyocytes contained the vector genome, suggesting minimal cardiac uptake of the viral vectors, which likely hindered the achievement of meaningful transgene expression. Potential solutions to this issue could be to increase the dose of recombinant AAV encoding the transgene, optimize the delivery method, or isolate AAV variants with enhanced tropism for cardiomyocytes.

Despite the advantages, AAVs also exhibit challenges. Although less immunogenic than AVs, AAVs can still trigger immune responses and be recognized by pre-existing neutralizing antibodies, especially at the capsid proteins. This may lead to challenges for dose repetition, particularly in therapies requiring long-term or repeated administration. This problem can be tackled by engineering novel capsid variants to reduce immunogenicity and evade pre-existing antibodies as well as enhance tissue specificity [70]. Another challenge is that AAVs have limited cargo capacity of ~ 4.7 kb, which can restrict their use for large genes or complex regulatory elements. To overcome this problem, dual-vector systems with two AAV vectors can be used to deliver large genes by splitting them into smaller fragments that reassemble in host cells [71]. In addition, AAVs can be used in combination with CRISPR technologies to deliver much smaller guide RNAs to a specific site in the gene promoter to modulate gene expression [72].

#### Lentiviruses

Lentiviruses (LVs) are a type of retroviruses derived from the human immunodeficiency virus (HIV). It has emerged as a powerful tool for gene therapy due to its ability to deliver and stably integrate genes into the cell genome [73].

LVs offer several advantages. Stable gene integration into the host genome ensures permanent gene expression in transduced cells, which is advantageous for diseases requiring long-term therapeutic effects, such as inherited disorders or chronic conditions [74]. LVs elicit relatively mild immune responses, reducing the likelihood of immune-mediated clearance and allowing for sustained therapeutic effects [75]. Further, LVs possess large cargo capacity of up to ~ 10 kb, which is larger compared to AVs and AAVs, which makes them suitable for delivering complex genes with their regulatory elements. Like AVs and AAVs, LVs can efficiently transduce proliferative as well as non-proliferative cells, which broadens their tropism across different tissues [73].

As LVs integrate their transgenes into the host genome, there is a risk of insertional mutagenesis, which can potentially activate oncogenes or disrupt tumor suppressor genes. This risk can be mitigated by using self-inactivating (SIN) vectors and insulator sequences [76]. LVs can also integrate into unintended sites in the genome, posing risks of off-target effects. In addition, although most viral genes are removed in LVs, residual sequences derived from HIV can provoke immune responses in some cases. To overcome these challenges, Integration-Deficient LVs are developed (IDLVs) may be a solution. It allows LVs to retain efficient transduction capacity, but deliver genes without permanent integration, reducing the risk of insertional mutagenesis [77]. This strategy transforms LVs to mimic AVs or AAVs to provide episomal gene expression for applications where permanent integration is not required. In addition, integration site targeting has also been developed to engineer LVs to preferentially integrate into safe harbor sites in the genome [78]. Lastly, capsid engineering has also been applied to improve tissue specificity and reduce immune recognition, enhancing safety and efficiency [79].

### Non-viral Vector

#### Lipid Nanoparticles

Lipid nanoparticles (LNPs) have emerged as a promising non-viral vector system for gene delivery. These lipid-based nanoparticles are composed of lipids that can encapsulate nucleic acids, including DNA, RNA, or small interfering RNA (siRNA), facilitating their delivery to target cells [80]. LNPs are also able to deliver genetic material to both dividing and non-dividing cells [81]. LNPs are considered safe and biocompatible due to their lipid-based composition, which is similar to the cell membrane structure. This reduces the risk of toxicity and adverse immune responses as compared to viral vectors [82]. Additionally, LNPs can be relatively easy to engineer to avoid immune system activation, allowing for repetitive dosing [83]. Unlike viral vectors, LNPs deliver genetic material without integration into the genome, which reduces the risk of disrupting essential genes or activating oncogenes, making them a safer alternative [84].

LNPs can be synthesized relatively easily and in large quantities, making them a cost-effective option for gene therapy, in comparison to viral vectors, which require complex manufacturing processes [85]. The scalability and reproducibility of LNPs production make them highly attractive for both preclinical and clinical applications. LNPs have demonstrated success as delivery vehicles for therapeutic applications such as siRNA against transthyretin for cardiac amyloidosis treatment and COVID-19 mRNA vaccines [86–88]. While LNPs are effective at delivering genetic material to a wide range of tissues, they exhibit low targeting specificity [89]. This may be encountered by modifying the surface properties, such as adding targeting ligands, to enhance specificity and reduce off-target effects [90]. Further, even though LNPs can carry a wide range of nucleic acids, they are still limited in their cargo size as compared to viral vectors [91]. This may restrict their ability to deliver large genes or complex gene constructs. LNPs are also susceptible to degradation over time, which can affect their ability to efficiently deliver their cargo [92].

#### Naked Oligonucleotides

Plasmids are the simplest form of vectors for the transportation of DNA into the nucleus, and they consist of 1,000–200,000 bp of a circular double-stranded DNA. Although most plasmids only contain one gene, it is possible to insert a polycistronic cassette for multiple gene expression [93]. Compared to other vectors such as viruses, plasmids have the advantage of being easy to produce and propagate while also displaying low immunogenicity. However, their transfection efficiency in the heart is low [94, 95]. To overcome this issue, there have been attempts to improve plasmid efficiency by using liposome-DNA complexes or microbubbles [96]. It is important to take into consideration that plasmid-based expression of lncRNAs is limited to their size.

Other forms of naked oligonucleotides are antisense oligonucleotides (ASO) and small interfering RNAs (siRNAs). While plasmids are double-stranded DNA vectors, ASOs are single-stranded DNA molecules with the ability to bind mRNAs thus silencing its expression by inducing RNAse-H mediated degradation of the target [97]. Several ASOs have been developed to target and treat cardiovascular diseases such as elevated LDL-cholesterol Levels, high Lipoprotein(a), and in total knee arthroplasty displaying low adverse events and in-site reactions [97, 98].

Finally, siRNAs are double-stranded RNA molecules of about 20–25 nucleotides in length, which function by interfering with mRNA expression. Once mature, the siRNA binds to the RISC complex and its most stable 5′ end integrates with the RISC complex and directs it to its target. The latter is then cleaved by the Argonaute family (Ago2). Similar to ASOs, siRNAs have also been tested in clinical settings to treat CVDs. In particular, siRNAs were tested to treat high LDL cholesterol, atherosclerotic CVD (ASCVD), and cardiac amyloidosis, some showing serious adverse events [99].

In summary, for viral vectors, AAVs are the more preferred vectors as multiple serotypes can be used with tissue-specific tropism and these rarely integrate into the genome. AAVs can persist in organs with non-dividing or slowly dividing cells for prolonged periods without adverse effects. However, these advantages come at the expense of a limited cargo size and response to pre-existing or newly developed neutralizing antibodies. LNPs as a non-viral delivery system were developed to overcome the limitations of the viral vectors. Their ability to deliver genes efficiently, coupled with their low immunogenicity and high scalability, positions them as an attractive option for clinical applications. However, LNPs show low tissue specificity and accumulate primarily in the liver, limiting effective delivery to the cardiovascular system. Similarly to LNPs, naked oligonucleotides also offer advantages such as ease of production, high scalability, and low immunogenicity. However, they suffer from limitations including low efficiency in the heart and limited size.

## Gene Therapy Strategies for lncRNAs

There are three gene therapy strategies for lncRNAs that are deemed efficient and are currently widely under investigation, namely siRNAs/shRNAs, ASOs, and CRISPR-Cas-based strategies (Table 3). Several lncRNAs have been knocked down using traditional siRNAs in cell lines [100]. However, in vivo experiments using siRNAs have been challenging, partly due to the lack of efficient delivery methods and the limited bioavailability of siRNAs in animals [101]. siRNAs target primarily cytoplasmic RNAs due to their mechanism of action, which requires RISC, localized in the cytoplasm. This limits the efficiency of this strategy to target RNAs residing in the nucleus. ASOs, on the other hand, can act equally efficiently in the cytoplasm and nucleus, as they act based on steric blocking [102].

**Table 3:** Potential strategies to target lncRNAs

Strategy	Target (molecule)	Primary Location of Action	Reversibility	Efficiency	Challenges	Clinical progress
siRNA/shRNA	RNA	Cytoplasm	Reversible	High knock-down	Off-target effects; immune activation; transient effect; saturation of RNAi machinery	NCT04832971 (phase 2), Inclisiran (FDA-EMA approved)
Steric ASOs	RNA (no cleavage)	Nucleus & cytoplasm	Reversible	Moderate–high (chemistry-dependent)	Target accessibility; requires chemical optimization; delivery to certain tissues	NCT03070782 (phase 2a), Golodirsen (FDA-EMA approved)
Gapmer ASOs	RNA (via RNase H1)	Nucleus	Reversible (long-lasting)	High (nuclear targets)	Limited cytoplasmic activity; off-target cleavage; immune activation; requires RNase H1	NCT00607373 (Phase 3), Inotersen (FDA-EMA approved)
CRISPR KO (Cas9)	Genomic DNA	Nucleus	Irreversible	High (requires clonal isolation)	Off-target mutations; clonal variation; low HDR efficiency; DNA damage response	NCT03872479 (CRISPR-Cas9 editing; early phase clincal trial), Casgevy (CRISPR-Cas9 KO; FDA-EMA approved)
CRISPRi (dCas9-KRAB)	Promoter/TSS DNA (repression)	Nucleus	Reversible	High (transcriptional repression)	Requires sustained expression; position-dependent sgRNA design; may not fully silence all genes	Not yet in clinial trials
CRISPRa (dCas9-VP64, dCas9-p300)	Promoter/TSS DNA (activation)	Nucleus	Reversible	Moderate–high (locus-dependent)	Ineffective on inaccessible promoters; requires sgRNA screening; risk of off-target activation	Not yet in clinial trials
Cas13	RNA	Nucleus & cytoplasm (variant-dependent)	Reversible	High for abundant transcripts	Collateral RNA cleavage (some variants); delivery of both Cas13 and sgRNA; limited in vivo experience	NCT06031727 (early phase 1)

ASOs show some limitations for clinical use, due to in vivo toxicity and the lack of suitable delivery methods, which prevents adequate tissue targeting by a given dose. To tackle this, ASOs are usually engineered to increase their affinity to the target RNA, improve resistance to degradation by nucleases, and reduce immunogenicity. These chemical modifications include GapmeR ASOs, a RNA–DNA–RNA single-stranded oligonucleotide possessing ribonucleotides that contain a 2′-O-methoxyethyl modified sugar backbone [103], with or without additional modifications such as locked nucleic acids (LNA) and S-constrained ethyl residues [104].

Different from general ASOs, Gapmers more effectively degrade RNA transcripts in the nucleus, given their action via RNAse-H1, which is mostly localized in the nuclear compartment. In addition to siRNAs and ASOs, CRISPR-Cas systems have shown promise for the modulation of lncRNAs at the DNA level. Various forms of CRISPR–Cas systems allow deletion (CRISPR–Cas9), inhibition (CRISPRi, CRISPR-dCas9-KRAB), or activation (CRISPRa, CRISPR-dCas9-VP64) of lncRNA genes [105]. Further, they can also be used to induce degradation of lncRNA transcripts (CRISPR–Cas13) [106]. Given the lack of functional open reading frames, targeting lncRNAs using CRISPR-Cas is more challenging than targeting protein-coding genes, which may delay the therapeutic application of this system in vivo.

## Challenges and Perspectives

Using non-coding RNAs as gene therapy targets holds great potential, not only because the number of non-coding genes exceeds the number of coding genes, thus greatly increasing the pool of potential target genes, but also because non-coding RNAs bypass the need for the translation machinery in the target cell. However, there are also several challenges when it comes to using long non-coding RNAs as gene therapeutics. One of those challenges is that the delivery methods, both viral and non-viral, have been primarily optimized for delivery of protein-coding genes. For instance, lentiviruses, which are RNA viruses, require the producer cells to transcribe RNA as a single large transcript per virus particle. Once inside target cells, this viral RNA needs to be reverse transcribed, incorporated in the genome, and transcribed to produce a therapeutic RNA. In the case of a protein coding gene, this RNA would contain an open reading frame that is translated exactly into the desired protein. In contrast, for non-coding RNAs, the transcribed RNA is already the therapeutic molecule, but it contains flanking RNA sequences (particularly on the 3’ side) needed for proper lentiviral function, such as the 3’LTR, which may hamper function of the non-coding RNA. These limitations are most troublesome when lncRNA levels need to be increased, whereas inhibition or degradation of lncRNAs is likely more feasible.

Non-viral RNA delivery systems, such as lipid nanoparticles (LNPs), offer alternatives, but they also present unique challenges. Stabilization of RNA cargoes typically requires chemical modifications—for instance, N1-methylpseudouridine, widely used in mRNA COVID-19 vaccines [107]. While such modifications enhance translational efficiency and reduce immunogenicity for protein-coding mRNAs, their impact on lncRNAs is less predictable. Because lncRNA function often depends on complex secondary or tertiary structures and RNA–protein interactions, indiscriminate modification may disrupt activity. A critical next step for translational research is to develop rational design strategies that map modifiable sites on therapeutic lncRNAs without impairing function. This will require more detailed structural and mechanistic studies of lncRNA biology, as well as improvements in RNA synthesis platforms capable of site-specific modifications. In parallel, delivery systems must be further optimized for cardiac applications. Targeting cardiomyocytes remains difficult, especially using LNPs, which often show liver tropism. Immunogenicity of viral vectors such as AAVs also remains a regulatory and clinical bottleneck, with pre-existing immunity and toxicity concerns limiting patient eligibility. Novel capsid engineering approaches and tissue-specific promoters may help overcome these barriers, but rigorous preclinical validation in large-animal models is needed.

Looking forward, clinical translation of lncRNA-based therapies for CVD will require coordinated advances on several fronts. First, a priority should be the development of lncRNA-specific delivery vectors—for example, lentiviral backbones that minimize or remove interfering 3’ elements, or LNP formulations tailored for cardiac tropism using peptide-based targeting ligands. Second, preclinical studies must move beyond rodent models and begin testing lncRNA therapeutics in large-animal models of heart failure or myocardial infarction, where pharmacokinetics, biodistribution, and efficacy can be evaluated more realistically. Third, lncRNA function must be mechanistically validated in the disease context. This includes identifying interaction partners, mapping critical secondary structures, and defining expression thresholds for therapeutic efficacy—all of which are essential for regulatory approval. Fourth, regulatory pathways for RNA-based therapies are still largely shaped by mRNA vaccine and siRNA precedents, and may not fully capture the unique challenges of lncRNA drugs. Future clinical trial designs will need to address uncertainties about long-term expression, tissue specificity, and off-target effects, especially for non-coding transcripts with partial homology to other RNAs. By addressing these technical and regulatory hurdles head-on, the field will be well-positioned to translate the promise of lncRNA therapeutics into meaningful treatments for cardiovascular disease.

## Data Availability

No data generated by the authors was used for this review paper.
